# The National Medical Journal

**Published:** 1872-02

**Authors:** 


### The National Medical Journal.

On account of a determination on the part of tbe publishers of this Journal to insert an article, which in the shape presented, was not deemed suitable by the editors, Drs. Busey and Lee, these gentlemen have retired from the charge of the Journal and the February No. will be issued under the entire control of the publishers.

:o:

